# Supplementary material for: Machine learning the dimension of a Fano variety
Source: Nat Commun. 2023 Sep 8;14:5526. doi: 10.1038/s41467-023-41157-1 (PMC10491608; doi:10.1038/s41467-023-41157-1)
Supplement: Supplementary file 1 — Supplementary Information [file 41467_2023_41157_MOESM1_ESM.pdf]

Machine Learning the Dimension of a Fano Variety: Supplementary Material

Tom Coates[†], Alexander M. Kasprzyk[‡], Sara Veneziale[*†]

Supplementary Notes

We begin with an introduction to weighted projective spaces and toric varieties, aimed at non-specialists.

Projective spaces and weighted projective spaces The fundamental example of a Fano variety is two-dimensional projective space $\mathbb{P}^2$. This is a quotient of $\mathbb{C}^3 \setminus \{0\}$ by the group $\mathbb{C}^\times$, where the action of $\lambda \in \mathbb{C}^\times$ identifies the points (x, y, z) and $(\lambda x, \lambda y, \lambda z)$ in $\mathbb{C}^3 \setminus \{0\}$. The variety $\mathbb{P}^2$ is smooth: we can see this by covering it with three open sets U_x, U_y, U_z that are each isomorphic to the plane $\mathbb{C}^2$:

$$\begin{aligned} U_x &= \{(1, Y, Z)\} && \text{given by rescaling } x \text{ to } 1 \\ U_y &= \{(X, 1, Z)\} && \text{given by rescaling } y \text{ to } 1 \\ U_z &= \{(X, Y, 1)\} && \text{given by rescaling } z \text{ to } 1 \end{aligned}$$

Here, for example, in the case U_x we take $x \neq 0$ and set $Y = y/x$, $Z = z/x$.

Although the projective space $\mathbb{P}^2$ is smooth, there are closely related Fano varieties called weighted projective spaces [1, 2] that have singularities. For example, consider the weighted projective plane $\mathbb{P}(1, 2, 3)$: this is the quotient of $\mathbb{C}^3 \setminus \{0\}$ by $\mathbb{C}^\times$, where the action of $\lambda \in \mathbb{C}^\times$ identifies the points (x, y, z) and $(\lambda x, \lambda^2 y, \lambda^3 z)$. Let us write

$$\mu_n = \{e^{2\pi k \mathtt{i}/n} \mid k \in \mathbb{Z}\}$$

for the group of nth roots of unity. The variety $\mathbb{P}(1, 2, 3)$ is once again covered by open sets

$$\begin{aligned} U_x &= \{(1, Y, Z)\} && \text{given by rescaling } x \text{ to } 1 \\ U_y &= \{(X, 1, Z)\} && \text{given by rescaling } y \text{ to } 1 \\ U_z &= \{(X, Y, 1)\} && \text{given by rescaling } z \text{ to } 1 \end{aligned}$$

[*]Corresponding author, s.veneziale21@imperial.ac.uk
[†]Department of Mathematics, Imperial College London, UK
[‡]School of Mathematical Sciences, University of Nottingham, UK

but this time we have $U_x \cong \mathbb{C}^2$, $U_y \cong \mathbb{C}^2/\mu_2$, and $U_z = \mathbb{C}^2/\mu_3$. This is because, for example, when we choose $\lambda \in \mathbb{C}^\times$ to rescale (x, y, z) with $z \neq 0$ to $(X, Y, 1)$, there are three possible choices for λ and they differ by the action of μ_3. In particular this lets us see that $\mathbb{P}(1, 2, 3)$ is singular. For example, functions on the chart $U_y \cong \mathbb{C}^2/\mu_2$ are polynomials in X and Z that are invariant under $X \mapsto -X$, $Z \mapsto -Z$, or in other words

$$\begin{aligned} U_y &= \operatorname{Spec} \mathbb{C}[X^2, XZ, Z^2] \\ &= \operatorname{Spec} \mathbb{C}[a, b, c]/(ac - b^2) \end{aligned}$$

Thus the chart U_y is the solution set for the equation $ac - b^2 = 0$, as pictured in Supplementary Figure 1a. Similarly, the chart $U_z \cong \mathbb{C}^2/\mu_3$ can be written as

$$\begin{aligned} U_z &= \operatorname{Spec} \mathbb{C}[X^3, XY, Y^3] \\ &= \operatorname{Spec} \mathbb{C}[a, b, c]/(ac - b^3) \end{aligned}$$

and is the solution set to the equation $ac - b^3 = 0$, as pictured in Supplementary Figure 1b. The variety $\mathbb{P}(1, 2, 3)$ has singular points at $(0, 1, 0) \in U_y$ and $(0, 0, 1) \in U_z$, and away from these points it is smooth.

There are weighted projective spaces of any dimension. Let $a_1, a_2, \ldots, a_N$ be positive integers such that any subset of size $N - 1$ has no common factor, and consider

$$\mathbb{P}(a_1, a_2, \ldots, a_N) = (\mathbb{C}^N \setminus \{0\})/\mathbb{C}^\times$$

where the action of $\lambda \in \mathbb{C}^\times$ identifies the points

$$(x_1, x_2, \ldots, x_N) \qquad \text{and} \qquad (\lambda^{a_1} x_1, \lambda^{a_2} x_2, \ldots, \lambda^{a_N} x_N)$$

in $\mathbb{C}^N \setminus \{0\}$. The quotient $\mathbb{P}(a_1, a_2, \ldots, a_N)$ is an algebraic variety of dimension $N - 1$. A general point of $\mathbb{P}(a_1, a_2, \ldots, a_N)$ is smooth, but there can be singular points. Indeed, $\mathbb{P}(a_1, a_2, \ldots, a_N)$ is covered by N open sets

$$U_i = \{(X_1, \ldots, X_{i-1}, 1, X_{i+1}, \ldots, X_N)\} \qquad i \in \{1, 2, \ldots, N\}$$

given by rescaling x_i to 1; here we take $x_i \neq 0$ and set $X_j = x_j/x_i$. The chart U_i is isomorphic to $\mathbb{C}^{N-1}/\mu_{a_i}$, where μ_{a_i} acts on $\mathbb{C}^{N-1}$ with weights a_j, $j \neq i$. In Reid's notation, this is the cyclic quotient singularity $\frac{1}{a_i}(a_1, \ldots, \widehat{a_i}, \ldots, a_N)$; it is smooth if and only if $a_i = 1$.

The topology of weighted projective space is very simple, with

$$H^k\left(\mathbb{P}(a_1, a_2, \ldots, a_N); \mathbb{Q}\right) = \begin{cases} \mathbb{Q} & \text{if } 0 \leq k \leq 2N - 2 \text{ and } k \text{ is even;} \\ 0 & \text{otherwise.} \end{cases}$$

Hence every weighted projective space has second Betti number $b_2 = 1$. There is a closed formula [3, Proposition D.9] for the regularized quantum period of $X = \mathbb{P}(a_1, a_2, \ldots, a_N)$:

$$\widehat{G}_X(t) = \sum_{k=0}^{\infty} \frac{(ak)!}{(a_1 k)!(a_2 k)! \cdots (a_N k)!} t^{ak} \tag{1}$$

where $a = a_1 + a_2 + \cdots + a_N$.

Toric varieties of Picard rank 2 As well as weighted projective spaces, which are quotients of $\mathbb{C}^N \setminus \{0\}$ by an action of $\mathbb{C}^\times$, we will consider varieties that arise as quotients of $\mathbb{C}^N \setminus S$ by $(\mathbb{C}^\times)^2$, where S is a union of linear subspaces. These are examples of *toric varieties* [4, 5]. Specifically, consider a matrix

$$\begin{pmatrix} a_1 & a_2 & \cdots & a_N \\ b_1 & b_2 & \cdots & b_N \end{pmatrix} \tag{2}$$

with non-negative integer entries and no zero columns. This defines an action of $(\mathbb{C}^\times)^2$ on $\mathbb{C}^N$, where $(\lambda, \mu) \in (\mathbb{C}^\times)^2$ identifies the points

$$(x_1, x_2, \ldots, x_N) \quad \text{and} \quad (\lambda^{a_1}\mu^{b_1}x_1, \lambda^{a_2}\mu^{b_2}x_2, \ldots, \lambda^{a_N}\mu^{b_N}x_N)$$

in $\mathbb{C}^N$. Set $a = a_1 + a_2 + \ldots + a_N$ and $b = b_1 + b_2 + \cdots + b_N$, and suppose that (a, b) is not a scalar multiple of (a_i, b_i) for any i. This determines linear subspaces

$$S_+ = \{(x_1, x_2, \ldots, x_N) \mid x_i = 0 \text{ if } b_i/a_i < b/a\}$$
$$S_- = \{(x_1, x_2, \ldots, x_N) \mid x_i = 0 \text{ if } b_i/a_i > b/a\}$$

of $\mathbb{C}^N$, and we consider the quotient

$$X = (\mathbb{C}^N \setminus S)/(\mathbb{C}^\times)^2 \tag{3}$$

where $S = S_+ \cup S_-$. See e.g. [6, §A.5].

These quotients behave in many ways like weighted projective spaces. Indeed, if we take the weight matrix (2) to be

$$\begin{pmatrix} a_1 & a_2 & \cdots & a_N & 0 \\ 0 & 0 & \cdots & 0 & 1 \end{pmatrix}$$

then X coincides with $\mathbb{P}(a_1, a_2, \ldots, a_N)$. We will consider only weight matrices such that the subspaces S_+ and S_- both have dimension two or more; this implies that the second Betti number $b_2(X) = 2$, and hence X is not a weighted projective space. We will refer to such quotients (3) as *toric varieties of Picard rank two*, because general theory implies that the Picard lattice of X has rank two. The dimension of X is $N-2$. As for weighted projective spaces, toric varieties of Picard rank two can have singular points, the precise form of which is determined by the weights (2). There is also a closed formula [3, Proposition C.2] for the regularized quantum period. Let C denote the cone in $\mathbb{R}^2$ defined by the equations $a_i x + b_i y \geq 0$, $i \in \{1, 2, \ldots, N\}$. Then

$$\widehat{G}_X(t) = \sum_{(k,l) \in \mathbb{Z}^2 \cap C} \frac{(ak + bl)!}{(a_1k + b_1l)!(a_2k + b_2l)! \cdots (a_Nk + b_Nl)!} t^{ak+bl} \tag{4}$$

Classification results Weighted projective spaces with terminal quotient singularities have been classified in dimensions up to four; see Supplementary Table 1 for a summary. There are 35 three-dimensional Fano toric varieties with terminal quotient singularities and Picard rank two [7]. There is no known classification of Fano toric varieties with terminal quotient singularities in higher dimension, even when the Picard rank is two.

Supplementary Methods 1

Data analysis: weighted projective spaces As discussed in the main text, we computed an initial segment $(c_0, c_1, \ldots, c_m)$ of the regularized quantum period, with $m \approx 100\,000$, for all the examples in the sample of 150 000 weighted projective spaces with terminal quotient singularities. We then considered $\{\log c_d\}_{d \in S}$ where $S = \{d \in \mathbb{Z}_{\geq 0} \mid c_d \neq 0\}$. To reduce dimension we fitted a linear model to the set $\{(d, \log c_d) \mid d \in S\}$ and used the slope and intercept of this model as features. The linear fit produces a close approximation of the data. Supplementary Figure 2 shows the distribution of the standard errors for the slope and the y-intercept: the errors for the slope are between 3.9×10^{-8} and 1.4×10^{-5}, and the errors for the y-intercept are between 0.0022 and 0.82. As we will see below, the standard error for the y-intercept is a good proxy for the accuracy of the linear model. This accuracy decreases as the dimension grows – see Supplementary Figure 2c – but we will see below that this does not affect the accuracy of the machine learning classification.

Data analysis: toric varieties of Picard rank 2 We fitted a linear model to the set $\{(d, \log c_d) \mid d \in S\}$ where $S = \{d \in \mathbb{Z}_{\geq 0} \mid c_d \neq 0\}$, and used the slope and intercept of this linear model as features. The distribution of standard errors for the slope and y-intercept of the linear model are shown in Supplementary Figure 3. The standard errors for the slope are small compared to the range of slopes, but in many cases the standard error for the y-intercept is relatively large. As Supplementary Figure 4 illustrates, discarding data points where the standard error s_{int} for the y-intercept exceeds some threshold reduces apparent noise. As discussed in the main text, we believe that this reflects inaccuracies in the linear regression caused by oscillatory behaviour in the initial terms of the quantum period sequence.

Example 1. Let us consider in more detail the toric variety from Example 3 in the main text. In Supplementary Figure 5 we plot $\log c_d$ along with its linear approximation. Supplementary Figure 5a shows only the first 250 terms, whilst Supplementary Figure 5b shows the interval between the 1000th and the 1250th term. We see considerable deviation from the linear approximation among the first 250 terms; the deviation reduces for larger d.

Supplementary Methods 2

We performed our experiments using scikit-learn [8], a standard machine learning library for Python. The computations that produced the data shown in Figure 7b of the main text were performed using Mathematica [9]. All code required to replicate the results in this paper is available from Bitbucket under an MIT license [10].

Weighted projective spaces We excluded dimensions one and two from the analysis, since there is only one weighted projective space in each case (namely $\mathbb{P}^1$ and $\mathbb{P}^2$). Therefore we have a dataset of 149 998 slope-intercept pairs, labelled by the dimension which varies between three and ten. We standardised the features, by translating

the means to zero and scaling to unit variance, and applied a Support Vector Machine (SVM) with linear kernel and regularisation parameter $C = 10$. By looking at different train–test splits we obtained the learning curves shown in Supplementary Figure 6. The figure displays the mean accuracies for both training and validation data obtained by performing five random test-train splits each time: the shaded areas around the lines correspond to the 1σ region, where σ denotes the standard deviation. Using 10% (or more) of the data for training we obtained an accuracy of 99.99%. In Supplementary Figure 7 we plot the decision boundaries computed by the SVM between neighbouring dimension classes.

Toric varieties of Picard rank 2 In light of the discussion above, we restricted attention to toric varieties with Picard rank two such that the y-intercept standard error s_{int} is less than 0.3. We also excluded dimension two from the analysis, since in this case there are only two varieties (namely, $\mathbb{P}^1 \times \mathbb{P}^1$ and the Hirzebruch surface $\mathbb{F}_1$). The resulting dataset contains 67 443 slope-intercept pairs, labelled by dimension; the dimension varies between three and ten, as shown in Supplementary Table 2.

Support Vector Machine We used a linear SVM with regularisation parameter $C = 50$. By considering different train–test splits we obtained the learning curves shown in Supplementary Figure 8, where the means and the standard deviations were obtained by performing five random samples for each split. Note that the model did not overfit. We obtained a validation accuracy of 88.2% using 70% of the data for training. Supplementary Figure 9 shows the decision boundaries computed by the SVM between neighbouring dimension classes. Supplementary Figure 10 shows the confusion matrices for the same train–test split.

Random Forest Classifier We used a Random Forest Classifier (RFC) with 1500 estimators and the same features (slope and y-intercept for the linear model). By considering different train–test splits we obtained the learning curves shown in Supplementary Figure 11; note again that the model did not overfit. Using 70% of the data for training, the RFC gave a validation accuracy of 89.4%. Supplementary Figure 12 shows confusion matrices for the same train–test split.

Feed-forward neural network As discussed in the main text, neural networks do not handle unbalanced datasets well, and therefore we removed the toric varieties with dimensions three, four, and five from our dataset: see Supplementary Table 2. We trained a Multilayer Perceptron (MLP) classifier on the same features, using an MLP with three hidden layers $(10, 30, 10)$, Adam optimiser [11], and rectified linear activation function [12]. Different train–test splits produced the learning curve in Supplementary Figure 13; again the model did not overfit. Using 70% of the data for training, the MLP gave a validation accuracy of 88.7%. One could further balance the dataset, by randomly undersampling so that there are the same number of representatives in each dimension (8244 representatives: see Supplementary Table 2). This resulted in a

slight decrease in accuracy: the better balance was outweighed by loss of data caused by undersampling.

Feed-forward neural network with many features We trained an MLP with the same architecture, but supplemented the features by including $\log c_d$ for $1 \leq d \leq 100$ (unless c_d was zero in which case we set that feature to zero), as well as the slope and y-intercept as before. We refer to the previous neural network as MLP_2, because it uses 2 features, and refer to this neural network as MLP_{102}, because it uses 102 features. Supplementary Figure 14 shows the learning curves obtained for different train–test splits. Using 70% of the data for training, the MLP_{102} model gave a validation accuracy of 97.7%.

We do not understand the reason for the performance improvement between MLP_{102} and MLP_2. But one possible explanation is the following. Recall that the first 1000 terms of the period sequence were excluded when calculating the slope and intercept, because they exhibit irregular oscillations that decay as d grows. These oscillations reduce the accuracy of the linear regression. The oscillations may, however, carry information about the toric variety, and so including the first few values of $\log(c_d)$ potentially makes more information available to the model. For example, examining the pattern of zeroes at the beginning of the sequence (c_d) sometimes allows one to recover the values of a and b – see Equation 5 in the main text for the notation. This information is relevant to estimating the dimension because, as a very crude approximation, larger a and b go along with larger dimension. Omitting the slope and intercept, however, and training on the coefficients $\log c_d$ for $1 \leq d \leq 100$ with the same architecture gave an accuracy of only 62%.

Comparison of models The validation accuracies of the SVM, RFC, and the neural networks MLP_2 and MLP_{102}, on the same data set ($s_{\mathrm{int}} < 0.3$, dimension between six and ten), are compared in Supplementary Table 3. Their confusion matrices are shown in Supplementary Table 4. All models trained on only the regression data performed well, with the RFC slightly more accurate than the SVM and the neural network MLP_2 slightly more accurate still. Misclassified examples are generally in higher dimension, which is consistent with the idea that misclassification is due to convergence-related noise. The neural network trained on the supplemented feature set, MLP_{102}, outperforms all other models. However, as discussed in the main text, feature importance analysis using SHAP values showed that the slope and the intercept were the most influential features in the prediction.

Supplementary Discussion

Comparison with Principal Component Analysis An alternative approach to dimensionality reduction, rather than fitting a linear model to $\log c_d$, would be to perform Principal Component Analysis (PCA) on this sequence and retain only the first few principal components. Since the vectors (c_d) have different patterns of zeroes – c_d is non-zero only if d is divisible by the Fano index r of X – we need to perform PCA

for Fano varieties of each index r separately. We analysed this in the weighted projective space case, finding that for each r the first two components of PCA are related to the growth coefficients (A, B) from Theorem 5 by an invertible affine-linear transformation. That is, our analysis suggests that the coefficients (A, B) contain exactly the same information as the first two components of PCA. Note, however, that the affine-linear transformation that relates PCA to (A, B) varies with the Fano index r. Using A and B as features therefore allows for meaningful comparison between Fano varieties of different index. Furthermore, unlike PCA-derived values, the coefficients (A, B) can be computed for a single Fano variety, rather than requiring a sufficiently large collection of Fano varieties of the same index.

Towards more general Fano varieties Weighted projective spaces and toric varieties of Picard rank two are very special among Fano varieties. It is hard to quantify this, because so little is known about Fano classification in the higher-dimensional and non-smooth cases, but for example this class includes only 18% of the $\mathbb{Q}$-factorial terminal Fano toric varieties in three dimensions. On the other hand, one can regard weighted projective spaces and toric varieties of Picard rank two as representative of a much broader class of algebraic varieties called toric complete intersections. Toric complete intersections share the key properties that we used to prove Theorems 5 and 6 – geometry that is tightly controlled by combinatorics, including explicit expressions for genus-zero Gromov–Witten invariants in terms of hypergeometric functions – and we believe that the rigorous results of this paper will generalize to the toric complete intersection case. All smooth two-dimensional Fano varieties and 92 of the 105 smooth three-dimensional Fano varieties are toric complete intersections [3]. Many theorems in algebraic geometry were first proved for toric varieties and later extended to toric complete intersections and more general algebraic varieties; cf. [13–15] and [16, 17].

The machine learning paradigm presented here, however, applies much more broadly. Since our models take only the regularized quantum period sequence as input, we expect that whenever we can calculate $\widehat{G}_X$ – which is the case for almost all known Fano varieties – we should be able to apply a machine learning pipeline to extract geometric information about X.

Supplementary Tables

Supplementary Table 1: **The classification of low-dimensional weighted projective spaces with terminal quotient singularities.**

Dimension			
1	2	3	4
$\mathbb{P}^1$	$\mathbb{P}^2$	7 cases<br>see [7]	28 686 cases<br>see [18]

Supplementary Table 2: **The distribution by dimension among toric varieties of Picard rank two in our dataset with $s_{\text{int}} < 0.3$.**

Rank-two toric varieties with $s_{\text{int}} < 0.3$		
Dimension	Sample size	Percentage
3	17	0.025
4	758	1.124
5	5 504	8.161
6	12 497	18.530
7	16 084	23.848
8	13 701	20.315
9	10 638	15.773
10	8 244	12.224
Total	67 443	

Supplementary Table 3: **Comparison of model accuracies.** Accuracies for various models applied to the dataset of toric varieties of Picard rank two and dimension at least six: a Support Vector Machine with linear kernel, a Random Forest Classifier, and the neural networks MLP_2 and MLP_{102}.

ML models			
SVM	RFC	MLP_2	MLP_{102}
87.7%	88.6%	88.7%	97.7%

Supplementary Table 4: **Comparison of confusion matrices.** Confusion matrices for various models applied to the dataset of toric varieties of Picard rank two and dimension at least six: a Support Vector Machine with linear kernel, a Random Forest Classifier, and the neural networks MLP_2 and MLP_{102}.

Model	True confusion matrix	Predicted confusion matrix

SVM

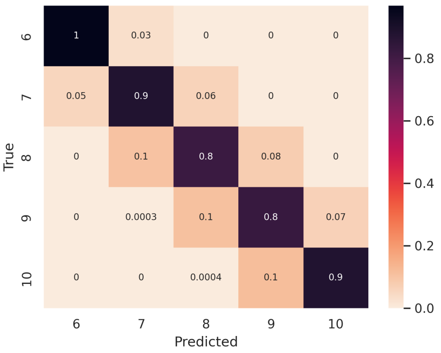

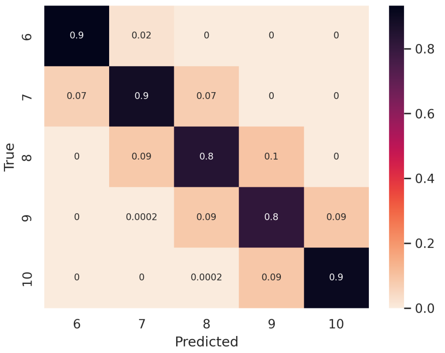

RFC

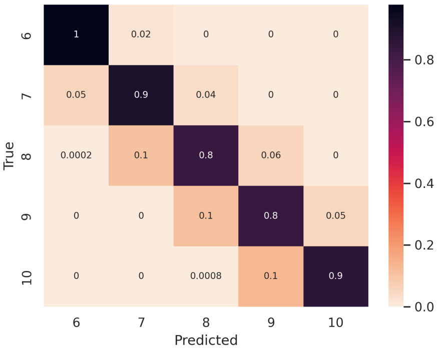

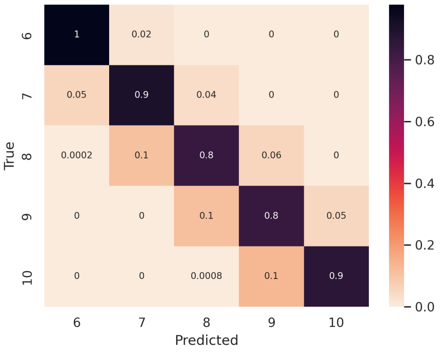

MLP_2

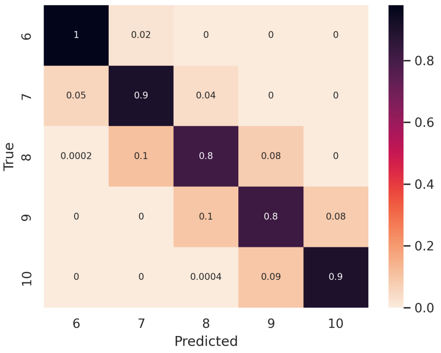

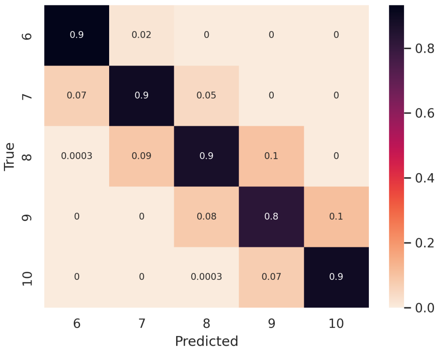

MLP_{102}

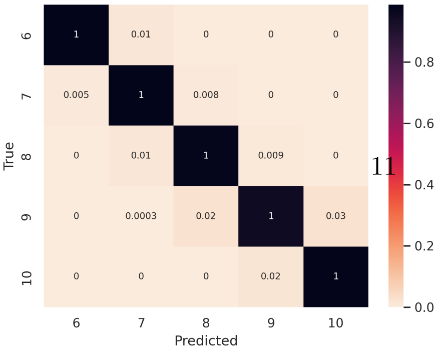

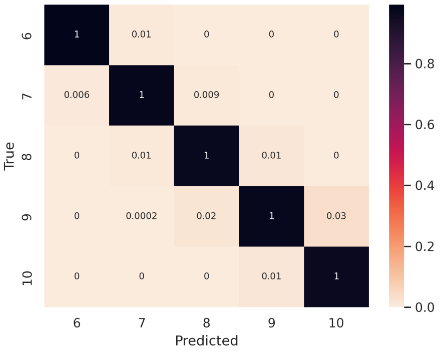

Supplementary Figures

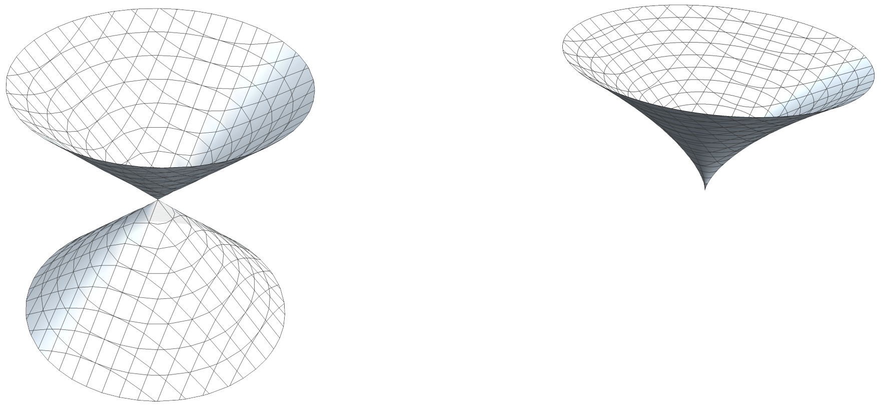

a b

Supplementary Figure 1: **Singular charts on the weighted projective space $\mathbb{P}(1,2,3)$:** (a) the real-valued points in the chart U_y. (b) the real-valued points in the chart U_z.

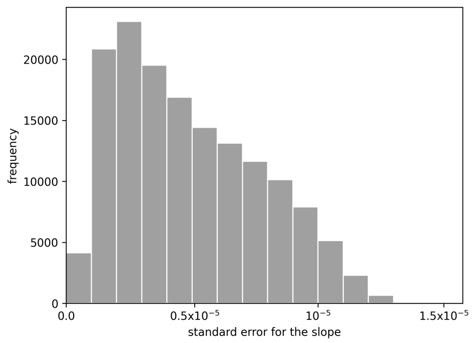

a

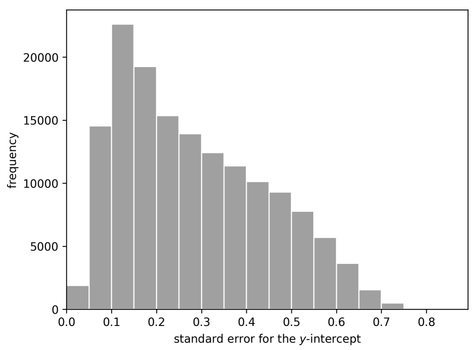

b

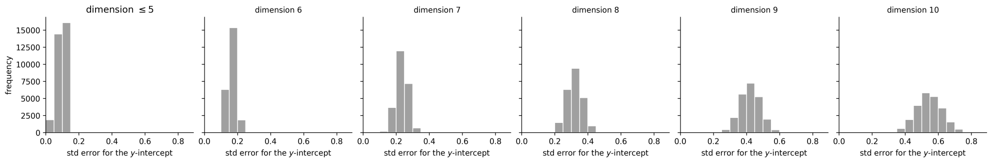

c

Supplementary Figure 2: **Standard errors for the slope and y-intercept.** The distribution of standard errors for the slope and y-intercept from the linear model applied to weighted projective spaces X with terminal quotient singularities: (a) standard error for the slope. (b) standard error for the y-intercept. (c) standard error for the y-intercept by dimension.

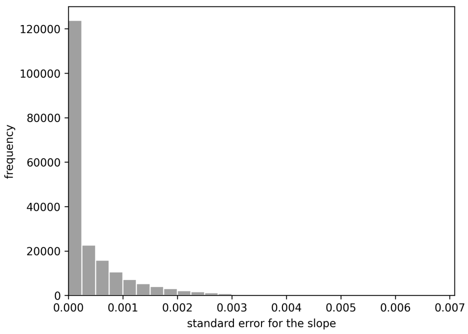

a

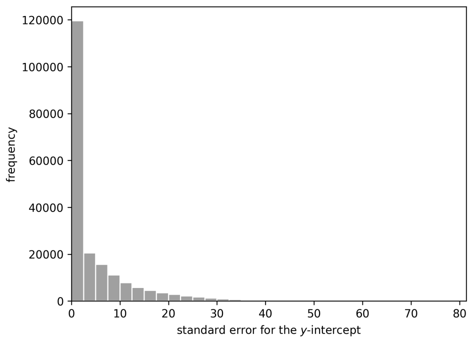

b

Supplementary Figure 3: **Standard errors for the slope and y-intercept.** The distribution of standard errors for the slope and y-intercept from the linear model applied to toric varieties of Picard rank two with terminal quotient singularities: (a) standard error for the slope. (b) standard error for the y-intercept.

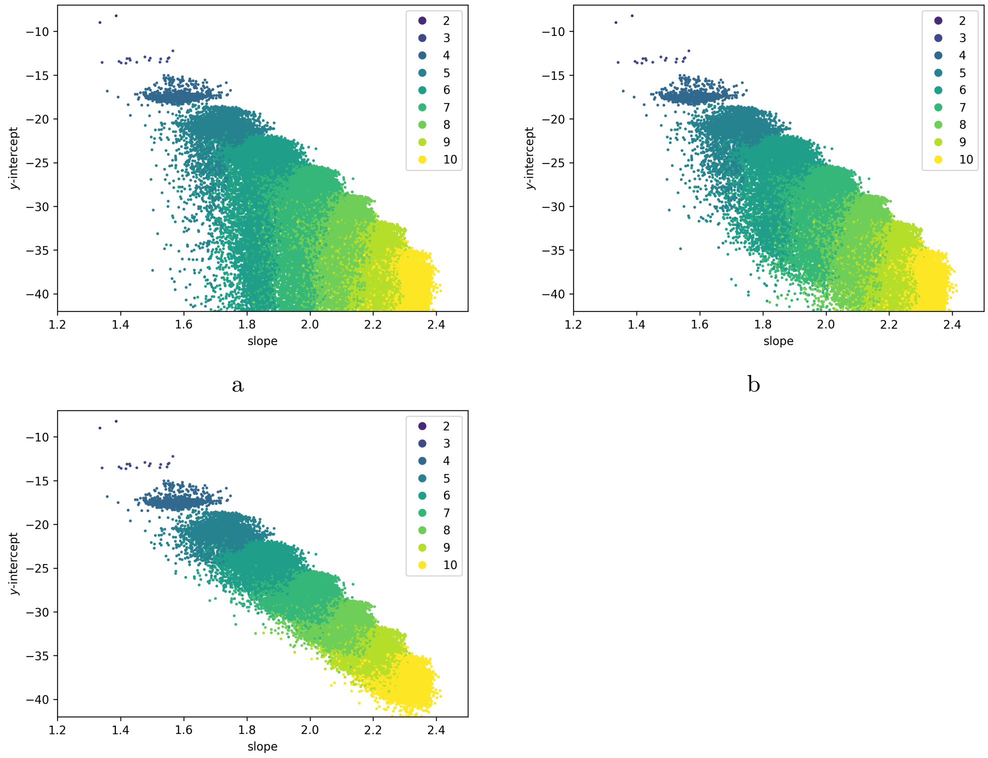

a

b

c

Supplementary Figure 4: **The slopes and y-intercepts from the linear model applied to toric varieties of Picard rank two with terminal quotient singularities.** Data points are selected according to the standard error s_{int} for the y-intercept. The colour records the dimension of the toric variety. (a) All data points. (b) Points with $s_{\mathrm{int}} < 1$: 101 183/200000 points. (c) Points with $s_{\mathrm{int}} < 0.3$: 67 445/200000 points.

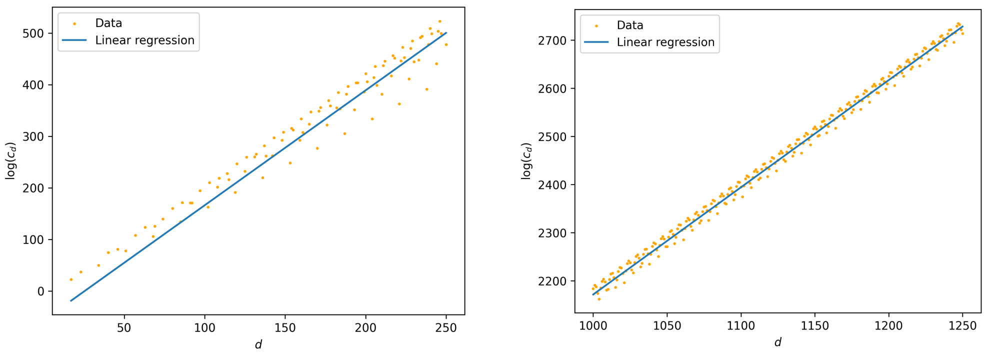

a

b

Supplementary Figure 5: **The logarithm of the non-zero coefficients c_d for Example 1:** (a) the first 250 terms. (b) terms between $d = 1000$ and $d = 1250$. In each case, the linear approximation is also shown.

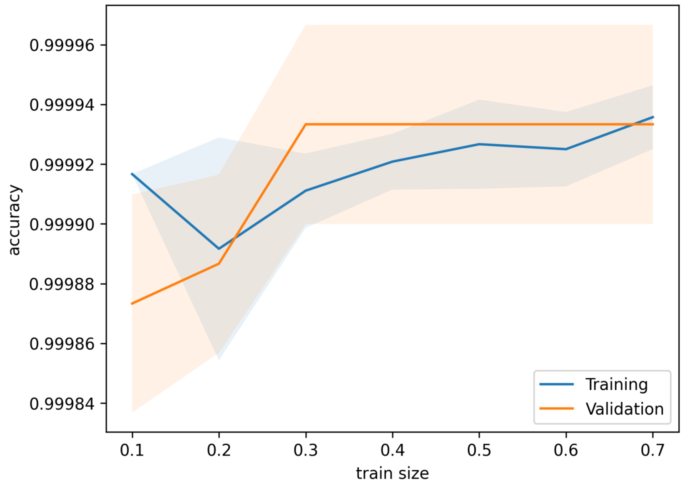

Supplementary Figure 6: **Learning curves for a Support Vector Machine with linear kernel applied to the dataset of weighted projective spaces.** The plot shows the means of the training and validation accuracies for five different random train–test splits. The shaded regions show the 1σ interval, where σ is the standard deviation.

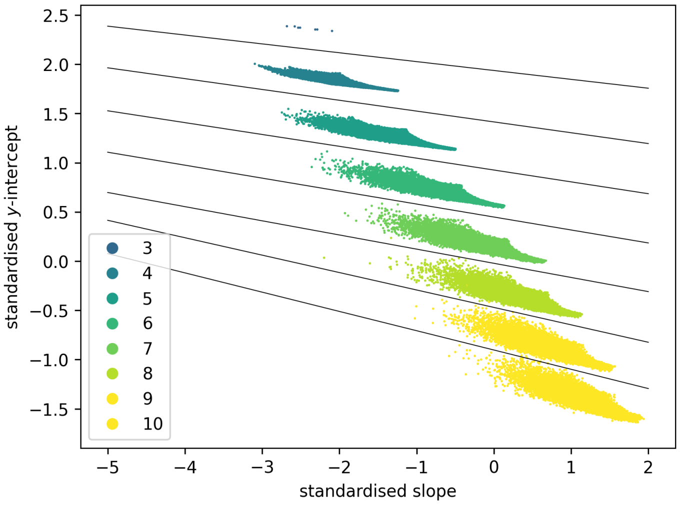

Supplementary Figure 7: **Decision boundaries computed from a Support Vector Machine with linear kernel trained on 70% of the dataset of weighted projective spaces.** Note that the data has been standardised.

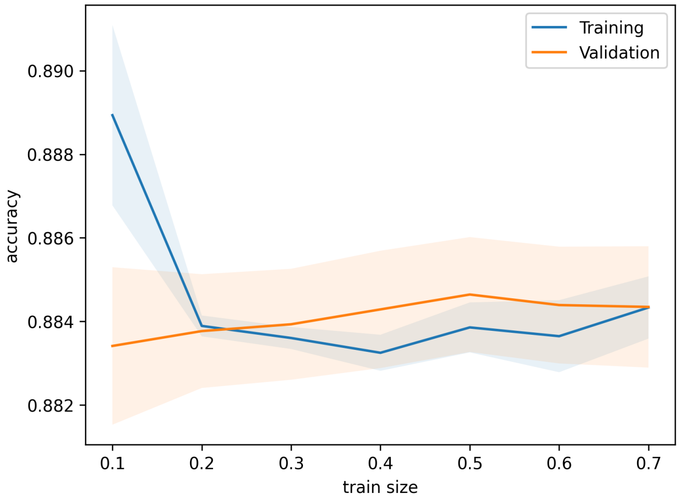

Supplementary Figure 8: **Learning curves for a Support Vector Machine with linear kernel applied to the dataset of toric varieties of Picard rank two.** The plot shows the means of the training and validation accuracies for five different random train–test splits. The shaded regions show the 1σ interval, where σ is the standard deviation.

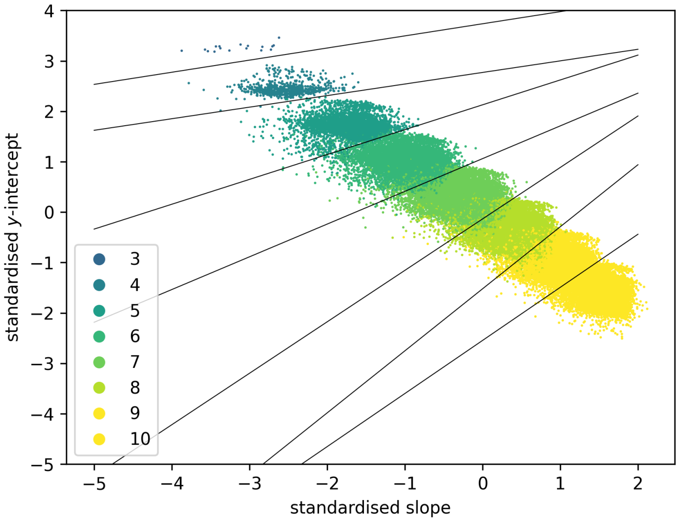

Supplementary Figure 9: **Decision boundaries computed from a Support Vector Machine with linear kernel trained on 70% of the dataset of toric varieties of Picard rank two.** Note that the data has been standardised.

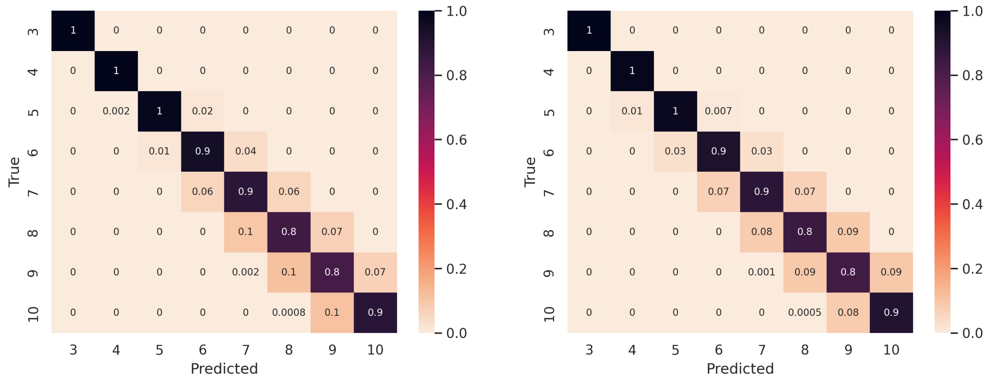

a Confusion matrix normalised with respect to the true values.

b Confusion matrix normalised with respect to the predicted values.

Supplementary Figure 10: **Confusion matrices for a Support Vector Machine with linear kernel trained on 70% of the dataset of toric varieties of Picard rank two.**

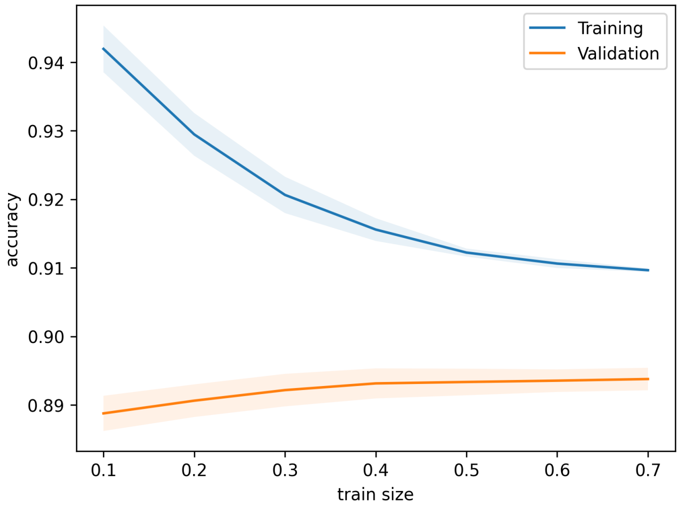

Supplementary Figure 11: **Learning curves for a Random Forest Classifier applied to the dataset of toric varieties of Picard rank two.** The plot shows the means of the training and validation accuracies for five different random train–test splits. The shaded regions show the 1σ interval, where σ is the standard deviation.

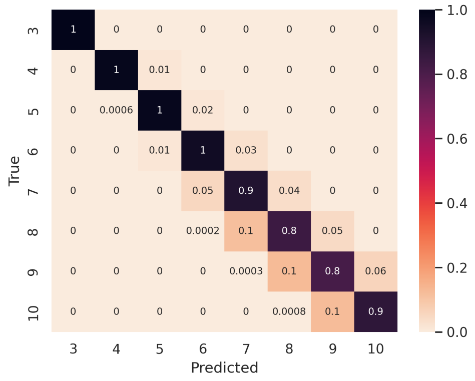

a Confusion matrix normalised with respect to the true values.

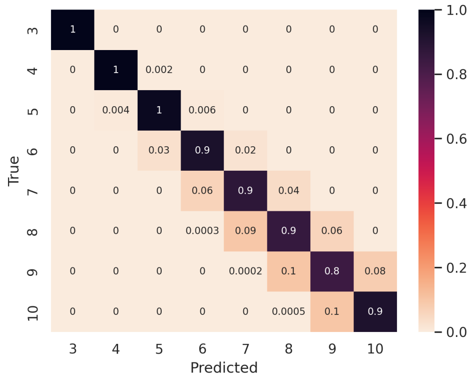

b Confusion matrix normalised with respect to the predicted values.

Supplementary Figure 12: **Confusion matrices for a Random Forest Classifier trained on 70% of the dataset of toric varieties of Picard rank two.**

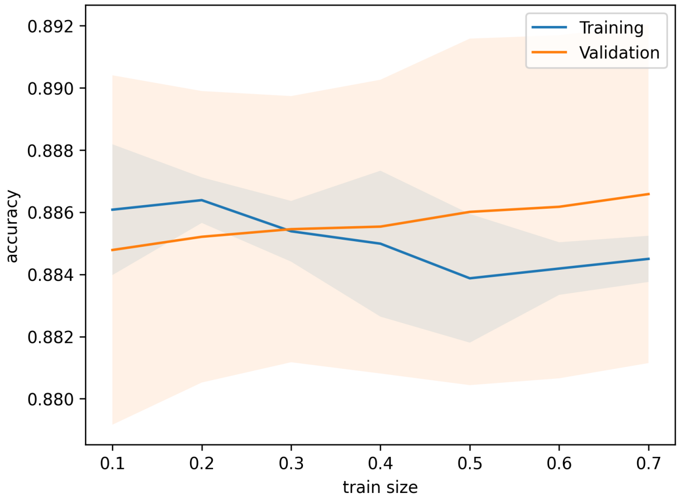

Supplementary Figure 13: **Learning curves for a Multilayer Perceptron classifier** MLP_2 **applied to the dataset of toric varieties of Picard rank two and dimension at least six, using just the regression data as features.** The plot shows the means of the training and validation accuracies for five different random train–test splits. The shaded regions show the 1σ interval, where σ is the standard deviation.

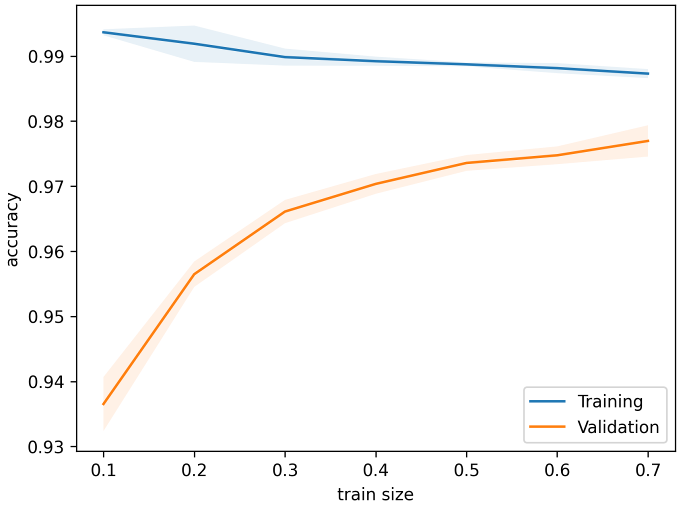

Supplementary Figure 14: **Learning curves for a Multilayer Perceptron classifier** MLP_{102} **applied to the dataset of toric varieties of Picard rank two and dimension at least six, using as features the regression data as well as** $\log c_d$ **for** $1 \leq d \leq 100$**.** The plot shows the means of the training and validation accuracies for five different random train–test splits. The shaded regions show the 1σ interval, where σ is the standard deviation.

Supplementary References

1. Dolgachev, I. in *Group actions and vector fields (Vancouver, B.C., 1981)* 34–71 (Springer, Berlin, 1982). doi:10.1007/BFb0101508.
2. Iano-Fletcher, A. R. in *Explicit birational geometry of 3-folds* 101–173 (Cambridge Univ. Press, Cambridge, 2000).
3. Coates, T., Corti, A., Galkin, S. & Kasprzyk, A. M. Quantum periods for 3-dimensional Fano manifolds. *Geom. Topol.* **20,** 103–256. doi:10.2140/gt.2016.20.103 (2016).
4. Fulton, W. *Introduction to toric varieties* The William H. Roever Lectures in Geometry, xii+157. doi:10.1515/9781400882526 (Princeton University Press, Princeton, NJ, 1993).
5. Cox, D. A., Little, J. B. & Schenck, H. K. *Toric varieties* xxiv+841. doi:10.1090/gsm/124 (American Mathematical Society, Providence, RI, 2011).
6. Brown, G., Corti, A. & Zucconi, F. in *The Fano Conference* 235–275 (Univ. Torino, Turin, 2004).
7. Kasprzyk, A. M. Toric Fano three-folds with terminal singularities. *Tohoku Math. J. (2)* **58,** 101–121. doi:10.2748/tmj/1145390208 (2006).
8. Pedregosa, F. *et al.* Scikit-learn: Machine Learning in Python. *Journal of Machine Learning Research* **12,** 2825–2830 (2011).
9. Wolfram Research, I. *Mathematica, Version 13.1* Champaign, IL, 2022. https://www.wolfram.com/mathematica.
10. Coates, T., Kasprzyk, A. M. & Veneziale, S. *Supporting code* https://bitbucket.org/fanosearch/mldim. 2022.
11. Kingma, D. P. & Ba, J. *Adam: A method for stochastic optimization* 2014. arXiv: 1412.6980 [cs.LG].
12. Agarap, A. F. *Deep learning using rectified linear units (ReLU)* 2018. arXiv: 1803.08375 [cs.NE].
13. Givental, A. B. Equivariant Gromov-Witten invariants. *Internat. Math. Res. Notices,* 613–663. doi:10.1155/S1073792896000414 (1996).
14. Givental, A. in *Topological field theory, primitive forms and related topics (Kyoto, 1996)* 141–175 (Birkhäuser Boston, Boston, MA, 1998).
15. Gross, M. & Siebert, B. *Intrinsic Mirror Symmetry* 2019. arXiv: 1909.07649 [math.AG].
16. Givental, A. B. Semisimple Frobenius structures at higher genus. *Internat. Math. Res. Notices,* 1265–1286. doi:10.1155/S1073792801000605 (2001).
17. Teleman, C. The structure of 2D semi-simple field theories. *Invent. Math.* **188,** 525–588. doi:10.1007/s00222-011-0352-5 (2012).
18. Kasprzyk, A. M. *Classifying terminal weighted projective space* 2013. arXiv: 1304.3029 [math.AG].
